# The Rise of Scoping Reviews in Nursing Science: Trends, Merits, and Responsible Use

**DOI:** 10.3390/nursrep15120423

**Published:** 2025-11-28

**Authors:** Richard Gray, Niall Higgins, Piyanee Yobas, Alessandro Stievano, Daniel Bressington

**Affiliations:** 1 School of Nursing and Midwifery, La Trobe University, Melbourne, VIC 3086, Australia; 2 School of Nursing, Queensland University of Technology, Brisbane, QLD 4059, Australia; niall.higgins@health.qld.gov.au; 3 Mental Health and Specialist Services, The Park, Wacol, QLD 4076, Australia; 4 Alice Lee Centre for Nursing Studies Yong Loo Lin School of Medicine, National University of Singapore, Singapore 117597, Singapore; nurpk@nus.edu.sg; 5 Nursing Professor Department of Clinical and Experimental Medicine, University of Messina, 98122 Messina, Italy; alessandro.stievano@gmail.com; 6 Faculty of Nursing, Chiang Mai University, Chiang Mai 50200, Thailand; daniel.bressington@cmu.ac.th

## 1. Introduction

Over the last 22 months (January 2024 through September 2025), *Nursing Reports* has published 649 documents, of which 116 (18%) are indexed in SCOPUS as literature reviews. Developing a deep understanding of the existing evidence is essential in building a sound scientific rationale for research, informing health policy and practice guidelines, and shaping clinical practice. Systematic reviews and meta-analyses, which aim to provide a comprehensive and unbiased synthesis of the available evidence [1], are therefore regarded as being at the highest level in the hierarchy of evidence [2]. However, some have argued that the perceived superiority of systematic reviews can result in products that are redundant or superficial, adding little to what the discipline already knows [3].

The fact that nearly one in five of the papers we publish (the situation likely similar in other leading journals in this discipline) are reviews is not necessarily a cause for concern. However, we have noticed a marked increase in scoping reviews submitted to *Nursing Reports*. Of the 116 reviews published over the last two years, 45 (39%) were scoping reviews (a full list is available in Supplementary Document S1), making this the most common form of literature review that we publish. This pattern reflects a broader trend toward technical review products, an approach that some believe risks prioritising procedural rigour over the type of critical engagement with the literature that truly advances scholarship [3]. Nursing, as a research discipline, appears to have embraced the scoping review. This trend is not unique to nursing; in medical education, the number of knowledge syntheses, including scoping reviews, has grown dramatically, with an overall increase of more than 2600% over the last two decades [4]. In this Editorial, we discuss the merits and limitations of scoping reviews and consider whether their domination is problematic for the discipline (spoiler: we think it is). Finally, we outline our editorial position on publishing this type of review.

## 2. Why Scoping Reviews? Origins and Intended Purpose

Scoping reviews emerged in response to concerns that traditional systematic reviews can be overly restrictive in terms of the types of evidence that they include. For example, most Cochrane systematic reviews focus exclusively on randomised controlled trials, potentially overlooking other sources of evidence that may be important and relevant. Systematic review methodology is also less suited to addressing broad or complex questions. To provide a more inclusive approach capable of mapping diverse forms of evidence, the scoping review was introduced in the mid-2000s. Their popularity in nursing and health research reflects the need for methods that can capture complexity and contextual variation across diverse study designs.

Read any scoping review and you will likely find two seminal papers cited—Arksey and O’Malley [5] and Levac et al. [6]—which laid the foundations for this approach. Arksey and O’Malley [5] are credited with introducing the initial framework, describing scoping reviews as a way to rapidly map key concepts in a research area and identify the main sources and types of evidence. Levac et al. [6] later refined this framework, emphasising that scoping reviews typically aim to examine the extent, range, and nature of research activity on a given topic or problem, assess whether a full systematic review is warranted, summarise the state of knowledge, and identify research gaps and directions for further enquiry. As Mays et al. [7] note, such mapping approaches are particularly valuable when the literature is diverse and complex, offering breadth rather than depth and avoiding the premature exclusion of relevant evidence. In essence, scoping reviews provide an overview of existing research, help justify the need for systematic reviews and meta-analysis, and highlight areas where further investigation is required.

## 3. Methodological Standards

Importantly, both the JBI (Joanna Briggs Institute), in 2014 [8], and PRISMA, in 2018 [9], have introduced reporting guidelines for scoping studies to improve consistency, transparency, and completeness in how these reviews are conducted and reported. The JBI guidance emphasises methodological rigour, including the clear articulation of objectives, comprehensive search strategies, and structured approaches to data charting and synthesis. PRISMA-ScR (Preferred Reporting Items for Systematic reviews and Meta-Analyses extension for Scoping Reviews) provides a 22-item checklist designed to ensure that authors report essential elements such as rationale, eligibility criteria, sources of evidence, and methods for data presentation. Together, these frameworks are designed to standardise reporting, reduce ambiguity, and enhance the reproducibility and utility of scoping reviews.

How does the scoping review differ from the traditional systematic review? We have summarised the key differences in Supplementary Table S1 for reference. In brief, scoping reviews are broad, exploratory, and iterative in nature. They are designed to map the extent, range, and nature of research activity and typically produce a narrative (qualitative) synthesis of what is known about a topic. Importantly, scoping reviews generally do not include a formal appraisal of the methodological quality of the studies included. In contrast, systematic reviews are prospectively designed to answer a focused question and generate a definitive (often quantitative) statement about the safety, effectiveness, and quality of evidence for a given intervention. Both approaches have value and contribute to knowledge development. However, scoping reviews should not be used as a basis for changing health policy or clinical practice. Systematic reviews, on the other hand, are intended to inform evidence-based decision-making and guide clinical practice and policy.

## 4. Trends in Nursing and Health Education: Are Scoping Reviews Dominating?

Several scoping reviews of scoping reviews have examined the quality of methodology and reporting [10,11,12]. Pham and colleagues [10] analysed 344 scoping reviews published between 1999 and 2012 and found substantial variability in conduct, including failure to use a recognised framework (e.g., JBI, PRISMA-SR), reliance on single-reviewer screening, limited expert input on search strategies, and poor reporting of data charting processes. Tricco and colleagues [11] similarly observed that most scoping reviews—8 out of 10—were undertaken to identify gaps in knowledge and recommend future research directions, yet few adhered to methodological guidance; only 13% reported a protocol, and stakeholder consultation was rare. In Medical Education, Maggio and colleagues [12] reviewed 101 scoping reviews and noted frequent misalignment between the stated rationale and research questions, incomplete compliance with PRISMA-SR reporting items, infrequent protocol publishing. Limited engagement with stakeholders was reported in only about one fifth of reviews [12] an observation that warrants further comment. The authors of the two seminal papers on scoping reviews [5,6], both underscore the value of consultation in enhancing the relevance and utility of findings. Arksey and O’Malley [5] suggest that involving stakeholders and people with lived experience can strengthen interpretation, while Levac et al. [6] argue that consultation should be considered an essential component of the process. We rarely, if ever, see evidence of stakeholder consultation in the scoping reviews we handle and a consider this is cause for concern. 

Why have scoping reviews in nursing science become so popular? One explanation is the perception that they are ‘easier’ to complete than systematic reviews (after all, they do not require critical appraisal or complex meta-analysis). However, this view is misleading. Scoping reviews can be demanding and resource-intensive. Developing a robust search strategy is often challenging, particularly in emerging areas where the terminology is inconsistent or poorly indexed and where broad review questions necessitate numerous search terms [11]. Such strategies can yield very large numbers of citations, requiring extensive screening (the average scoping review includes 118 studies [11] compared to just 14 studies in a typical systematic review [13]). The heterogeneity of included studies—in terms of both design and content—adds further complexity to data charting and synthesis, making these steps far from straightforward [14].

## 5. Our Internal Analysis of Nursing Reports (2024–2025)

We examined 45 scoping reviews published in *Nursing Reports* over the last year (see Supplementary Document S1). Collectively, these reviews included 1748 documents. The median number of included studies was 14. Compared with other disciplines, nursing scoping reviews appear to synthesise far fewer outputs. Does this raise the concern, as others have argued [3,15], that we are producing a proliferation of scoping reviews that add little value and contribute minimally to knowledge? Ioannidis [15] has warned that the mass production of unnecessary, misleading, and conflicted reviews risks transforming evidence synthesis into a ‘publication factory’, serving academic or commercial interests rather than promoting evidence-based practice. This difference may reflect narrower review scopes, resource constraints, or methodological preferences within nursing research, but it also underscores the need for critical reflection on the purpose and utility of these reviews.

## 6. Overinterpretation and Methodological Overreach

Looking back over the scoping reviews we have published, we recognise a recurring issue: authors often overinterpret their findings and extend well beyond the methodological boundaries of a scoping review to make policy and clinical practice recommendations. Two examples—both published in *Nursing Reports*—illustrate this point. First, Ventura-Silva et al. [16] conducted a scoping review on the application of artificial intelligence in the organisation of nursing care. The authors included just ten papers (four surveys, three methodological papers, and three reviews) yet concluded that ‘AI represents a valuable contribution to the organisation of nursing care and should be used as a complement to nursing practice to support quick and accurate decision-making, especially in complex clinical situations’ [emphasis ours] [16]. A second example is Aling et al. [17], who reviewed barriers and facilitators for nurse–patient dialogue on sexuality and sexual health. Their review included nineteen studies (nine surveys, one mixed-methods, and nine qualitative) and concluded that ‘a norm-critical approach in education and practice will promote awareness and criticism of norms and power structures that result in exclusion within healthcare’ [again, our emphasis]. In both these examples, the authors move from mapping evidence to making declarative recommendations, steps that go far beyond the intended purpose of a scoping review. Scoping reviews are designed to map the breadth of evidence, not to determine effectiveness or prescribe practice, as they typically lack the critical appraisal and synthesis required for evidence-based recommendations. This is not an isolated phenomenon; similar examples abound in the nursing literature. It is a serious concern because such recommendations may be used to justify practice changes without robust evidence, potentially exposing patients to interventions that are untested, ineffective, or even harmful.

Scoping reviews are valuable for mapping evidence, identifying gaps, and determining whether a full systematic review is warranted. Yet nursing scholarship increasingly produces scoping reviews on narrow topics that synthesise few studies and add little new knowledge. This trend risks diverting efforts away from systematic reviews that inform practice. More concerning is the tendency for authors to overstep methodological boundaries by making prescriptive recommendations based solely on scoping findings. Although a scoping review of nursing scoping reviews might be needed confirm these concerns. 

## 7. Common Pitfalls and How to Avoid Them

To improve rigour and transparency, authors should avoid frequent missteps that undermine the quality of scoping reviews (see Box 1). Do not make prescriptive clinical or policy recommendations; keep findings descriptive and within the method’s remit. Publish or register a protocol (e.g., OSF) and document any iterative changes. Ensure transparent (including published studies, grey literature, reference lists, key journals), reproducible searches by reporting full strategies, dates, and limits, ideally co-developed with an information specialist. Use duplicate screening and data charting and report calibration or agreement metrics. Align the rationale, objectives, and *Population–Concept–Context* question, avoiding causal or “effectiveness” language. If critical appraisal is included, clarify its descriptive role in mapping rather than effect estimation. Design and report stakeholder consultation, specifying who was involved, how engagement occurred, and its influence on interpretation. Finally, comply with PRISMA-ScR by submitting the completed checklist and flow diagram with your manuscript.

Box 1 **Common pitfalls checklist for scoping reviews.**

☐ Avoid prescriptive clinical/policy recommendations; keep claims descriptive and within guidelines.

☐ Publish or register a protocol (e.g., OSF) and document iterative changes.

[Footnote: OSF (Open Science Framework) is a free platform for registering protocols and sharing materials. It provides a timestamped public record, especially useful for scoping reviews where PROSPERO may not apply.]

☐ Ensure transparent searches with full strategies (databases, dates, limits), ideally co-developed with an information specialist.

☐ Use duplicate screening and data charting; report calibration/agreement metrics.

☐ Align rationale, objectives, and PCC questions using an appropriate framework such as PCC, PEO, SPIDER, or SPICE. While PCC is commonly used in scoping reviews, other frameworks may be more suitable depending on the research context and objectives. Avoid causal or ‘effectiveness’ language.

[Footnote: PCC refers to Population–Concept–Context, a framework used to structure scoping review questions. It supports broad and exploratory inquiry, aligned with the method’s purpose. Other frameworks such as PEO, SPIDER, or SPICE may also be appropriate depending on the research context.]

☐ If critical appraisal is included, clarify its descriptive role in mapping and assessing risk of bias, rather than estimating effect size or intervention impact.

☐ Design and report stakeholder consultation (who, how, influence on interpretation).

☐ Comply with PRISMA-ScR: submit the completed checklist and include a flow diagram.

☐ If AI tools are used (e.g., for citation screening or data extraction), describe how they were implemented, validated, and agreed upon by the team. Establishing concordance at the outset ensures consistency and transparency in AI-assisted processes.

## 8. Stakeholder Engagement: Why and How

Another persistent issue is a lack of stakeholder engagement. In our experience, and as recent evidence confirms, engagement with people with lived experience and with communities more broadly is rarely undertaken in nursing research [18,19]. This omission undermines the relevance and impact of reviews. Stakeholder engagement matters because it improves the credibility, ethical rigour, and real-world applicability of research, ensuring that findings reflect the priorities and needs of those most affected. Stakeholder consultation is not optional; it is fundamental in producing scoping reviews that are relevant, credible, and ethically sound. Levac et al. [6] emphasise that consultation should be considered an essential component because it strengthens interpretation and ensures that findings reflect real-world priorities. However, published reviews often report engagement superficially or omit it entirely, risking tokenism and undermining trust. To address this, authors should (1) identify stakeholders early such as patients, carers, frontline nurses, educators, managers, and policy makers; (2) use initial consultation to refine *Population–Concept–Context* questions and inclusion criteria; (3) involve an information specialist to validate search terminology with stakeholders; (4) plan proportionate engagement through advisory panels, workshops, or interviews, seeking ethics approval where required; (5) chart consultation inputs separately to study data and clearly show how they influenced interpretation; (6) address equity, diversity, and inclusion by ensuring accessibility and, where appropriate, compensation. The JBI guidance reinforces these principles, highlighting the importance of meaningful engagement and living evidence approaches to improve applicability and impact. While stakeholder consultation is strongly encouraged, we acknowledge that for some authors, particularly those conducting their first scoping review or working outside established research teams, setting up patient and public involvement and engagement may be challenging. In such cases, proportionate engagement should be considered, and authors are encouraged to transparently report any limitations. In contexts where patient and public involvement and engagement is not yet standard practice, even small steps toward inclusion can be meaningful. Framing engagement in terms of its potential impact may support broader adoption and recognition [20].

## 9. Editorial Guidance and Submission Checklist (Box 2)

### Editorial Stance

Scoping reviews are welcome in *Nursing Reports* when they map evidence, clarify concepts, or identify gaps. They must not present prescriptive clinical or policy recommendations or imply effectiveness without a subsequent systematic review or meta-analysis. Manuscripts must demonstrate PRISMA-ScR compliance and justify why scoping and not another synthesis was the appropriate choice.

Box 2 **Submission checklist for scoping reviews.**

☐ Rationale for scoping review

Explain why this method fits the purpose and why a systematic review was not appropriate.

☐ Protocol link

Provide a registered or published protocol (e.g., OSF) and note any amendments.

☐ PCC-framed questions/objectives

Ensure alignment with scoping methodology; avoid causal or “effectiveness” language.

☐ Search transparency

Include full strategies (databases, dates, limits) and justify grey literature sources.

☐ Duplicate screening and charting

Describe methods, training, and agreement metrics.

☐ Stakeholder engagement details

Report who was involved, how engagement occurred, and its influence on interpretation.

☐ Synthesis remains descriptive

Present counts, themes, and gaps; do not make prescriptive recommendations.

☐ PRISMA-ScR compliance

Attach the completed checklist and include a flow diagram.

☐ Limitations and next steps

State the boundaries of the evidence and whether a systematic review is warranted.

## 10. Conclusions

Going forward, *Nursing Reports* will require scoping review submissions to justify the choice of method, demonstrate adherence to PRISMA-ScR and JBI guidance, and avoid prescriptive recommendations; manuscripts that fail to meet these standards may be rejected without external peer review. Scoping reviews play an important role in nursing scholarship when used to map evidence, clarify concepts, and identify gaps. However, their growing dominance, particularly when focused on narrow topics with few included studies, risks diluting the evidence base and diverting efforts from systematic reviews that inform practice and policy. The misuse of scoping reviews to make prescriptive recommendations further undermines methodological integrity and patient safety. To address these concerns, we urge authors to adhere to PRISMA-ScR and JBI guidance, embed meaningful stakeholder engagement, and keep conclusions within the intended scope of the method. Reviewers and editors must enforce these standards to ensure that scoping reviews complement rather than replace systematic reviews that underpin evidence-informed nursing care.

## Supplementary Materials

The following supporting information can be downloaded at: https://www.mdpi.com/article/10.3390/nursrep15120423/s1, Supplementary Document S1: scoping reviews published in *Nursing Reports*; Table S1*,* Supplementary Table S1, Key differences between scoping and systematic reviews.
