# Supplementary material for: The Rise of Scoping Reviews in Nursing Science: Trends, Merits, and Responsible Use
Source: Nurs Rep. 2025 Nov 28;15(12):423. doi: 10.3390/nursrep15120423 (PMC12735857; doi:10.3390/nursrep15120423)
Supplement: Supplementary file 1 [file nursrep-15-00423-s001.zip › nursrep-4013399-supplementary.pdf]

Supplementary document 1, list of scoping reviews published in Nursing Reports (2024 through October 2025)

Scopus
EXPORT DATE: 30 October 2025

1.
Kociolek, J., Gengo, R., Chiang-Hanisko, L.
AUTHOR FULL NAMES: Kociolek, Judyta (58287192600); Gengo, Rita De Cassia (57222993873); Chiang-Hanisko, Lenny (15830889900)
58287192600; 57222993873; 15830889900
Caring-Healing Modalities for Emotional Distress and Resilience in Persons with Cancer: A Scoping Review
(2025) Nursing Reports, 15 (9), art. no. 334
DOI: 10.3390/nursrep15090334
https://www.scopus.com/inward/record.uri?eid=2-s2.0-105017426724&doi=10.3390%2Fnursrep15090334&partnerID=40&md5=a295fd48e20a42f6aaed0c61acf314ac

DOCUMENT TYPE: Review
OPEN ACCESS: ALL OPEN ACCESS; GOLD OPEN ACCESS; GREEN ACCEPTED OPEN ACCESS; GREEN OPEN ACCESS

Number of included studies: 16

2.
Correia, P., Bernardes, R.A., Caldeira, S.
AUTHOR FULL NAMES: Correia, Patrícia (60116325900); Bernardes, Rafael A. (57208656236); Caldeira, Sílvia Maria Alves (55487193800)
60116325900; 57208656236; 55487193800
Instruments for Assessing Nursing Care Quality: A Scoping Review
(2025) Nursing Reports, 15 (9), art. no. 342
DOI: 10.3390/nursrep15090342
https://www.scopus.com/inward/record.uri?eid=2-s2.0-105017168916&doi=10.3390%2Fnursrep15090342&partnerID=40&md5=994c0f62ee0fd6085c967fa509731197

DOCUMENT TYPE: Review
OPEN ACCESS: ALL OPEN ACCESS; GOLD OPEN ACCESS; GREEN ACCEPTED OPEN ACCESS; GREEN OPEN ACCESS

Number of included studies: 45

3.

Vailati, A., Marcomini, I., Di Niquilo, M., Poliani, A., Rosa, D., Villa, G., Manara, D.F.
AUTHOR FULL NAMES: Vailati, Asia (60116431000); Marcomini, Ilaria (57223038007); Di Niquilo, Martina (60116218500); Poliani, Andrea (58312728300); Rosa, Debora (57204830687); Villa, Giulia (54584457800); Manara, Duilio Fiorenzo (6603110582)
60116431000; 57223038007; 60116218500; 58312728300; 57204830687; 54584457800; 6603110582
Beyond Care: A Scoping Review on the Work Environment of Oncology Nurses
(2025) Nursing Reports, 15 (9), art. no. 324
DOI: 10.3390/nursrep15090324
https://www.scopus.com/inward/record.uri?eid=2-s2.0-105017150555&doi=10.3390%2Fnursrep15090324&partnerID=40&md5=6fa5ab9d277609dd1cbac2f72f9dfb67

DOCUMENT TYPE: Review
OPEN ACCESS: ALL OPEN ACCESS; GOLD OPEN ACCESS; GREEN ACCEPTED OPEN ACCESS; GREEN OPEN ACCESS

Number of included studies: 20

4.
Bozzetti, M., Guberti, M., Lo Cascio, A., Privitera, D., Genna, C., Rodelli, S., Turchini, L., Amatucci, V., Giordano, L.N., Mora, V., Napolitano, D., Caruso, R.
AUTHOR FULL NAMES: Bozzetti, Mattia (57222344674); Guberti, Monica (55805801800); Lo Cascio, Alessio (57984356200); Privitera, Daniele (57216923320); Genna, Catia (58312453000); Rodelli, Silvia (60091523400); Turchini, Laura (57211106536); Amatucci, Valeria (57216894272); Giordano, Luciana Nicola (58923314700); Mora, Vincenzina (57203530618); Napolitano, Daniele (57211110962); Caruso, Rosario (24176763900)
57222344674; 55805801800; 57984356200; 57216923320; 58312453000; 60091523400; 57211106536; 57216894272; 58923314700; 57203530618; 57211110962; 24176763900
Uncovering the Professional Landscape of Clinical Research Nursing: A Scoping Review with Data Mining Approach
(2025) Nursing Reports, 15 (8), art. no. 266
DOI: 10.3390/nursrep15080266
https://www.scopus.com/inward/record.uri?eid=2-s2.0-105015561557&doi=10.3390%2Fnursrep15080266&partnerID=40&md5=ec3fb8af05d2c9e2a132cbd7eccc0fbd

DOCUMENT TYPE: Review
OPEN ACCESS: ALL OPEN ACCESS; GOLD OPEN ACCESS; GREEN ACCEPTED OPEN ACCESS; GREEN OPEN ACCESS

Number of included studies: 128

5.
Jobst, S., Kugler, C., Rebafka, A.

AUTHOR FULL NAMES: Jobst, Stefan (57189591689); Kugler, Christiane (57203070488); Rebafka, Anne (56667523000)
57189591689; 57203070488; 56667523000
Aspects of Self-Management After Solid Organ Transplantation—A Scoping Review
(2025) Nursing Reports, 15 (8), art. no. 304
DOI: 10.3390/nursrep15080304
https://www.scopus.com/inward/record.uri?eid=2-s2.0-105014481639&doi=10.3390%2Fnursrep15080304&partnerID=40&md5=21f05be63a2111fa1c8be18df30fd333

DOCUMENT TYPE: Review
OPEN ACCESS: ALL OPEN ACCESS; GOLD OPEN ACCESS; GREEN ACCEPTED OPEN ACCESS; GREEN OPEN ACCESS

Number of included studies: 742

6.
Pantaleo, F., Stievano, A., Mastroianni, C., Petrucci, G., Mazzitelli, N., Piredda, M., De Marinis, M.G., Marchetti, A.
AUTHOR FULL NAMES: Pantaleo, Flavia (57801074000); Stievano, Alessandro (6508016784); Mastroianni, Chiara (7006560892); Petrucci, Giorgia (57377753900); Mazzitelli, Natascia (57204863479); Piredda, Michela (22836193100); De Marinis, Maria Grazia Razia (22834503100); Marchetti, Anna (56651842000)
57801074000; 6508016784; 7006560892; 57377753900; 57204863479; 22836193100; 22834503100; 56651842000
The Nursing Student Licensure Examination: A Scoping Review
(2025) Nursing Reports, 15 (8), art. no. 299
DOI: 10.3390/nursrep15080299
https://www.scopus.com/inward/record.uri?eid=2-s2.0-105014476393&doi=10.3390%2Fnursrep15080299&partnerID=40&md5=75db7bd31e491c3d5039297fb635cc95

DOCUMENT TYPE: Review
OPEN ACCESS: ALL OPEN ACCESS; GOLD OPEN ACCESS; GREEN ACCEPTED OPEN ACCESS; GREEN OPEN ACCESS

Number of included studies: 23

7.
Assunção, A.S.L., Cunha, L.D.M.
AUTHOR FULL NAMES: Assunção, Adriana Sofia Lucas (60075073100); Cunha, Lara Daniela Matos (57225142315)
60075073100; 57225142315

Barriers and Facilitators to the Use of Capnography for Respiratory Monitoring by Nurses in Phase I Post-Anesthesia Care Unit: A Scoping Review
(2025) Nursing Reports, 15 (8), art. no. 292
DOI: 10.3390/nursrep15080292
https://www.scopus.com/inward/record.uri?eid=2-s2.0-105014396739&doi=10.3390%2Fnursrep15080292&partnerID=40&md5=88c7509de59d04c0d6a4a9a857aca0c9

DOCUMENT TYPE: Review
OPEN ACCESS: ALL OPEN ACCESS; GOLD OPEN ACCESS; GREEN ACCEPTED OPEN ACCESS; GREEN OPEN ACCESS

Number of included studies: 7

8.
Cucci, F., Marasciulo, D., Romani, M., Soldano, G., Cascio, D., De Nunzio, G., Caldararo, C., Rubbi, I., Vitale, E., Lupo, R., Conte, L.
AUTHOR FULL NAMES: Cucci, Federico (57428430100); Marasciulo, Dario (60075238100); Romani, Mattia (60074589900); Soldano, Giovanni (60075562600); Cascio, Donato (35619975800); De Nunzio, Giorgio (6603335538); Caldararo, Cosimo (57217228206); Rubbi, Ivan (55430913000); Vitale, Elsa (24342133600); Lupo, Roberto (57189011384); Conte, Luana (56911960400)
57428430100; 60075238100; 60074589900; 60075562600; 35619975800; 6603335538; 57217228206; 55430913000; 24342133600; 57189011384; 56911960400
The Contribution of Artificial Intelligence in Nursing Education: A Scoping Review of the Literature
(2025) Nursing Reports, 15 (8), art. no. 283
DOI: 10.3390/nursrep15080283
https://www.scopus.com/inward/record.uri?eid=2-s2.0-105014373133&doi=10.3390%2Fnursrep15080283&partnerID=40&md5=b65abdcdd216ac316ef1a19c69e1bcbd

DOCUMENT TYPE: Review
OPEN ACCESS: ALL OPEN ACCESS; GOLD OPEN ACCESS; GREEN ACCEPTED OPEN ACCESS; GREEN OPEN ACCESS

Number of included studies: 11

9.
El Arab, R.A., Al Moosa, O.A., Albahrani, Z., Alkhalil, I., Somerville, J., Abuadas, F.
AUTHOR FULL NAMES: El Arab, Rabie Adel (57204688079); Al Moosa, Omayma Abdulaziz (59727649300); Albahrani, Zahraa (60074854200); Alkhalil, Israa (60075667000); Somerville, Joel G. (58244259300); Abuadas, Fuad Hamdi (57193453829)
57204688079; 59727649300; 60074854200; 60075667000; 58244259300; 57193453829
Integrating Artificial Intelligence into Perinatal Care Pathways: A Scoping Review of Reviews of Applications, Outcomes, and Equity

(2025) Nursing Reports, 15 (8), art. no. 281
DOI: 10.3390/nursrep15080281
https://www.scopus.com/inward/record.uri?eid=2-s2.0-105014365042&doi=10.3390%2Fnursrep15080281&partnerID=40&md5=2208c6dd25775a8495de3b7d09278e59

DOCUMENT TYPE: Review
OPEN ACCESS: ALL OPEN ACCESS; GOLD OPEN ACCESS; GREEN ACCEPTED OPEN ACCESS; GREEN OPEN ACCESS

Number of included studies: 39

10.
Okpalauwaekwe, U., Franks, H., Kuo, Y.-F., Raji, M.A., Passy, E., Tzeng, H.-M.
AUTHOR FULL NAMES: Okpalauwaekwe, Udoka (57206755213); Franks, Hannah E. (59196579000); Kuo, Y. F. (7403230584); Raji, Mukaila Ajiboye (7003507730); Passy, Elise (59197001100); Tzeng, Huey Ming (hm) (7004870067)
57206755213; 59196579000; 7403230584; 7003507730; 59197001100; 7004870067
What Helps or Hinders Annual Wellness Visits for Detection and Management of Cognitive Impairment Among Older Adults? A Scoping Review Guided by the Consolidated Framework for Implementation Research
(2025) Nursing Reports, 15 (8), art. no. 295
DOI: 10.3390/nursrep15080295
https://www.scopus.com/inward/record.uri?eid=2-s2.0-105014355769&doi=10.3390%2Fnursrep15080295&partnerID=40&md5=70bdbace74ad166c78161e14d7832050

DOCUMENT TYPE: Review
OPEN ACCESS: ALL OPEN ACCESS; GOLD OPEN ACCESS; GREEN ACCEPTED OPEN ACCESS; GREEN OPEN ACCESS

Number of included studies: 19

11.
Lobão, C., Coelho, A., Parola, V., Neves, H., Pereira Sousa, J., Gonçalves, R.
AUTHOR FULL NAMES: Lobão, Catarina Alexandra Rodrigues Faria (57842806500); Coelho, Adriana Raquel Neves (57191226568); Parola, Vitor Sergio Oliveira (57191221098); Neves, Hugo Leiria (57203322454); Pereira Sousa, Joana (57210926095); Gonçalves, Rui Filipe Lopes (57541904600)
57842806500; 57191226568; 57191221098; 57203322454; 57210926095; 57541904600
Technostress in Nursing Education: A Scoping Review
(2025) Nursing Reports, 15 (7), art. no. 248
DOI: 10.3390/nursrep15070248

https://www.scopus.com/inward/record.uri?eid=2-s2.0-105011487202&doi=10.3390%2Fnursrep15070248&partnerID=40&md5=622be4faa0df78b021f4bce7037df0be

DOCUMENT TYPE: Review

Number of included studies: 3

12.
Sheikoleslami, R.L., Princeton, D.M., Mihaila Hansen, L.I., Kisa, S., Goyal, A.R.
AUTHOR FULL NAMES: Sheikoleslami, Rohangez Lida (59966233400); Princeton, Daisy Michelle (57267462900); Mihaila Hansen, Linda Iren (59965556300); Kisa, Sezer (14030019900); Goyal, Alka Rani (57190954138)
59966233400; 57267462900; 59965556300; 14030019900; 57190954138
Examining Factors Associated with Attrition, Strategies for Retention Among Undergraduate Nursing Students, and Identified Research Gaps: A Scoping Review
(2025) Nursing Reports, 15 (6), art. no. 182
DOI: 10.3390/nursrep15060182
https://www.scopus.com/inward/record.uri?eid=2-s2.0-105009309287&doi=10.3390%2Fnursrep15060182&partnerID=40&md5=9a52d2076e27b1f0957cc9c334eb7228

DOCUMENT TYPE: Review
OPEN ACCESS: ALL OPEN ACCESS; GOLD OPEN ACCESS; GREEN ACCEPTED OPEN ACCESS; GREEN OPEN ACCESS

Number of included studies: 19

13.
Chaichana, J., Eley, R., Watling, C., Ng, L.
AUTHOR FULL NAMES: Chaichana, Jiranut (59965283100); Eley, Robert M. (7003285815); Watling, Christopher N. (35076739900); Ng, Linda C. (56026144300)
59965283100; 7003285815; 35076739900; 56026144300
Evaluating Eye Tracking Technology in Nursing Education: A Scoping Review on Medication Administration Training
(2025) Nursing Reports, 15 (6), art. no. 185
DOI: 10.3390/nursrep15060185
https://www.scopus.com/inward/record.uri?eid=2-s2.0-105009309078&doi=10.3390%2Fnursrep15060185&partnerID=40&md5=f2a9cc2f3fc5d107060cf77151739cfd

DOCUMENT TYPE: Review
OPEN ACCESS: ALL OPEN ACCESS; GOLD OPEN ACCESS; GREEN ACCEPTED OPEN ACCESS; GREEN OPEN ACCESS

Number of included studies: 10

14.
Costa, P., Pereira Sousa, J., Nascimento, T., Cruchinho, P., Nunes, E., Gaspar, F., Lucas, P.
AUTHOR FULL NAMES: Costa, Patrícia (59556614200); Pereira Sousa, Joana (57210926095); Nascimento, Tiago (57222124063); Cruchinho, Paulo (58118727200); Nunes, Elisabete Maria Garcia Teles (57194719898); Gaspar, Maria Filomena Mendes (56099744200); Lucas, Pedro Ricardo Martins Bernardes (57218794026)
59556614200; 57210926095; 57222124063; 58118727200; 57194719898; 56099744200; 57218794026
Leadership Development in Undergraduate Nursing Students: A Scoping Review
(2025) Nursing Reports, 15 (5), art. no. 160
DOI: 10.3390/nursrep15050160
https://www.scopus.com/inward/record.uri?eid=2-s2.0-105006421754&doi=10.3390%2Fnursrep15050160&partnerID=40&md5=c4e01b5f589a86b5e8ceee4013173a5e

DOCUMENT TYPE: Review
OPEN ACCESS: ALL OPEN ACCESS; GOLD OPEN ACCESS; GREEN ACCEPTED OPEN ACCESS; GREEN OPEN ACCESS

Number of included studies: 25

15.
Alves, J., Azevedo, R., Marques, A., Encarnação, R., Alves, P.
AUTHOR FULL NAMES: Alves, José (59751589200); Azevedo, Rita (58960886800); Marques, Ana (59751589300); Encarnação, Rúben Miguel Câmara (57225146613); Alves, Paulo Jorge Pereira (57222710330)
59751589200; 58960886800; 59751589300; 57225146613; 57222710330
Pressure Injury Prediction in Intensive Care Units Using Artificial Intelligence: A Scoping Review
(2025) Nursing Reports, 15 (4), art. no. 126
DOI: 10.3390/nursrep15040126
https://www.scopus.com/inward/record.uri?eid=2-s2.0-105003500814&doi=10.3390%2Fnursrep15040126&partnerID=40&md5=f9aa54797507512bd0922e5f0f6555e1

DOCUMENT TYPE: Review
OPEN ACCESS: ALL OPEN ACCESS; GOLD OPEN ACCESS; GREEN ACCEPTED OPEN ACCESS; GREEN OPEN ACCESS

Number of included studies: 14

16.
da Costa, A.I.L.D., Barros, L., Diogo, P.

AUTHOR FULL NAMES: da Costa, Ana Inês Lourenço (58222728200); Barros, Luísa (56434611300); Diogo, Paula Manuela Jorge (6507294442)
58222728200; 56434611300; 6507294442
Emotional Labor in Pediatric Palliative Care: A Scoping Review
(2025) Nursing Reports, 15 (4), art. no. 118
DOI: 10.3390/nursrep15040118
https://www.scopus.com/inward/record.uri?eid=2-s2.0-105003497555&doi=10.3390%2Fnursrep15040118&partnerID=40&md5=1b87f6fd20bd35ebfd03fe9a597c8b44

DOCUMENT TYPE: Review
OPEN ACCESS: ALL OPEN ACCESS; GOLD OPEN ACCESS; GREEN ACCEPTED OPEN ACCESS; GREEN OPEN ACCESS

Number of included studies: 11

17.
Hohashi, N., Yi, Q.
AUTHOR FULL NAMES: Hohashi, Naohiro (6505851762); Yi, Qinqiuzi (57206403828)
6505851762; 57206403828
The Effectiveness of Family Group Conferencing and the Challenges to Its Implementation: A Scoping Review
(2025) Nursing Reports, 15 (4), art. no. 122
DOI: 10.3390/nursrep15040122
https://www.scopus.com/inward/record.uri?eid=2-s2.0-105003463418&doi=10.3390%2Fnursrep15040122&partnerID=40&md5=a068f0240f8b61d455a0190bfcac5f58

DOCUMENT TYPE: Review
OPEN ACCESS: ALL OPEN ACCESS; GOLD OPEN ACCESS; GREEN ACCEPTED OPEN ACCESS; GREEN OPEN ACCESS

Number of included studies: 26

18.
Machado, D.R., Brás, M.M., Almeida, A.L.D., Vilela, C.
AUTHOR FULL NAMES: Machado, Dora Ribeiro (59140800500); Brás, Manuel Alberto Morais (37096670900); Almeida, Assunção Laranjeira (59141000900); Vilela, Carlos (59751883400)
59140800500; 37096670900; 59141000900; 59751883400
The Relationship Between Nurses’ Emotional Competence and Evidence-Based Nursing: A Scoping Review
(2025) Nursing Reports, 15 (4), art. no. 124
DOI: 10.3390/nursrep15040124

https://www.scopus.com/inward/record.uri?eid=2-s2.0-105003458681&doi=10.3390%2Fnursrep15040124&partnerID=40&md5=6b7f53bbbacc14f8c630db5649640582

DOCUMENT TYPE: Review
OPEN ACCESS: ALL OPEN ACCESS; GOLD OPEN ACCESS; GREEN ACCEPTED OPEN ACCESS; GREEN OPEN ACCESS

Number of included studies: 11

19.
Picoito, R., Manuel, T., Vieira, S., Azevedo, R., Nunes, E., Alves, P.
AUTHOR FULL NAMES: Picoito, Ricardo Jorge De Barros Romeira (58679964400); Manuel, Tania (57553441400); Vieira, Sofia (59492541700); Azevedo, Rita (58960886800); Nunes, Elisabete Maria Garcia Teles (57194719898); Alves, Paulo Jorge Pereira (57222710330)
58679964400; 57553441400; 59492541700; 58960886800; 57194719898; 57222710330
Recommendations and Best Practices for the Risk Assessment of Pressure Injuries in Adults Admitted to Intensive Care Units: A Scoping Review
(2025) Nursing Reports, 15 (4), art. no. 128
DOI: 10.3390/nursrep15040128
https://www.scopus.com/inward/record.uri?eid=2-s2.0-105003437999&doi=10.3390%2Fnursrep15040128&partnerID=40&md5=290950ade3a5b61b484aa07cd9f99a86

DOCUMENT TYPE: Review
OPEN ACCESS: ALL OPEN ACCESS; GOLD OPEN ACCESS; GREEN ACCEPTED OPEN ACCESS; GREEN OPEN ACCESS

Number of included studies: 15

20.
Li, S., Craig, S., Mitchell, G., Fitzsimons, D., Creighton, L., Thompson, G., Stark, P.
AUTHOR FULL NAMES: Li, Shuangshuang (59713031100); Craig, Stephanie (58286820400); Mitchell, Gary (55641165800); Fitzsimons, D. (57203953034); Creighton, Laura (58776862200); Thompson, Gareth (57215590095); Stark, Patrick (7101748174)
59713031100; 58286820400; 55641165800; 57203953034; 58776862200; 57215590095; 7101748174
Nurse-Led Strategies for Lifestyle Modification to Control Hypertension in Older Adults: A Scoping Review
(2025) Nursing Reports, 15 (3), art. no. 106
DOI: 10.3390/nursrep15030106
https://www.scopus.com/inward/record.uri?eid=2-s2.0-105001268966&doi=10.3390%2Fnursrep15030106&partnerID=40&md5=83f06937bbb62b81f07160faf061d7b9

DOCUMENT TYPE: Review
OPEN ACCESS: ALL OPEN ACCESS; GOLD OPEN ACCESS; GREEN ACCEPTED OPEN ACCESS; GREEN OPEN ACCESS

Number of included studies: 5

21.
Longo, D., Ramacciati, N., Giusti, G.D.
AUTHOR FULL NAMES: Longo, Donato (57430671200); Ramacciati, Nicola (54917165100); Giusti, Gian Domenico (55257160300)
57430671200; 54917165100; 55257160300
Nurse Specialist in the Organ and Tissue Donation Process with Coordination Role: A Scoping Review
(2025) Nursing Reports, 15 (2), art. no. 39
DOI: 10.3390/nursrep15020039
https://www.scopus.com/inward/record.uri?eid=2-s2.0-85219179316&doi=10.3390%2Fnursrep15020039&partnerID=40&md5=fa6b8253bb678aab1bc44ece1cc62752

DOCUMENT TYPE: Review
OPEN ACCESS: ALL OPEN ACCESS; GOLD OPEN ACCESS; GREEN ACCEPTED OPEN ACCESS; GREEN OPEN ACCESS

Number of included studies: 43

22.
Valencia-Contrera, M., Rivera-Rojas, F., Villa-Velásquez, J., Cancino-Jiménez, D., Vallejos-Vergara, S., Febré, N.
AUTHOR FULL NAMES: Valencia-Contrera, Miguel Andrez (57715260300); Rivera-Rojas, Flérida M. (57200389708); Villa-Velásquez, Jenifer (58105260100); Cancino-Jiménez, Daniella (58680226500); Vallejos-Vergara, Solange (59497357700); Febré, Naldy Pamela Pamela (6603354603)
57715260300; 57200389708; 58105260100; 58680226500; 59497357700; 6603354603
Scoping Review on Ethical Considerations in Research on the Work–Family Interaction Process
(2025) Nursing Reports, 15 (2), art. no. 31
DOI: 10.3390/nursrep15020031
https://www.scopus.com/inward/record.uri?eid=2-s2.0-85218885715&doi=10.3390%2Fnursrep15020031&partnerID=40&md5=2828980b14b7ef1f60e0bd6c5327d6b9

DOCUMENT TYPE: Review
OPEN ACCESS: ALL OPEN ACCESS; GOLD OPEN ACCESS; GREEN FINAL OPEN ACCESS; GREEN OPEN ACCESS

Number of included studies: 10

23.
Gouveia, M., Morgado, T., Costa, T., Sampaio, F., Rosa, A., Sequeira, C.
AUTHOR FULL NAMES: Gouveia, Marta (58161549900); Morgado, Tânia Manuel Moço (57225148445); Costa, Tiago Filipe Oliveira (57219039294); Sampaio, Francisco Miguel Correia (56072757600); Rosa, Amorim Gabriel Santos (57225149241); Sequeira, Carlos Alberto da Cruz (55504544300)
58161549900; 57225148445; 57219039294; 56072757600; 57225149241; 55504544300
Intervention Programmes for First-Episode Psychosis: A Scoping Review
(2025) Nursing Reports, 15 (1), art. no. 16
DOI: 10.3390/nursrep15010016
https://www.scopus.com/inward/record.uri?eid=2-s2.0-85216129614&doi=10.3390%2Fnursrep15010016&partnerID=40&md5=5ef4923d7bc2b8bc9e086aef9bcea82e

DOCUMENT TYPE: Review
OPEN ACCESS: ALL OPEN ACCESS; GOLD OPEN ACCESS; GREEN ACCEPTED OPEN ACCESS; GREEN OPEN ACCESS

Number of included studies: 47

24.
Finlay, S., Anderson, T., Henderson, E., Brown Wilson, C.B., Stark, P., Carter, G., Rodger, M., Mihalis Doumas, M., O'Shea, E., Creighton, L., Craig, S., Crooks, S., Gillis, A., Mitchell, G.
AUTHOR FULL NAMES: Finlay, Stacey Louise (58020566800); Anderson, Tara (58777654300); Henderson, Elizabeth (59362200000); Brown Wilson, C. (14120647200); Stark, Patrick (7101748174); Carter, Gillian (57202610071); Rodger, Matthew W.M. (47661704900); Mihalis Doumas, Michail (9736344300); O'Shea, Emma (58520342800); Creighton, Laura (58776862200); Craig, Stephanie (58286820400); Crooks, Sophie (56890848600); Gillis, Arnelle (58959980300); Mitchell, Gary (55641165800)
58020566800; 58777654300; 59362200000; 14120647200; 7101748174; 57202610071; 47661704900; 9736344300; 58520342800; 58776862200; 58286820400; 56890848600; 58959980300; 55641165800
A Scoping Review of Educational and Training Interventions on Parkinson’s Disease for Staff in Care Home Settings
(2025) Nursing Reports, 15 (1), art. no. 20
DOI: 10.3390/nursrep15010020
https://www.scopus.com/inward/record.uri?eid=2-s2.0-85215690301&doi=10.3390%2Fnursrep15010020&partnerID=40&md5=00eb90e37d2a17c38cf424ad77c619d7

DOCUMENT TYPE: Review
OPEN ACCESS: ALL OPEN ACCESS; GOLD OPEN ACCESS; GREEN ACCEPTED OPEN ACCESS; GREEN OPEN ACCESS

Number of included studies: 7

25.
MacLean, R., Durepos, P., Keeping-Burke, L., Witherspoon, R., Morris, P., Gibbons, C., Taylor, N., McCloskey, R.
AUTHOR FULL NAMES: MacLean, Rachel (58312990800); Durepos, Pamela (57193169660); Keeping-Burke, Lisa (55566387600); Witherspoon, Richelle L. (57218606163); Morris, Patricia (57219039030); Gibbons, Caroline (57098887300); Taylor, Natasha C. (58312592400); McCloskey, Rose M. (55566217200)
58312990800; 57193169660; 55566387600; 57218606163; 57219039030; 57098887300; 58312592400; 55566217200
Education and Training on Infection Prevention and Control Provided by Long-Term Care Homes to Visitors: A Scoping Review
(2025) Nursing Reports, 15 (1), art. no. 17
DOI: 10.3390/nursrep15010017
https://www.scopus.com/inward/record.uri?eid=2-s2.0-85215661509&doi=10.3390%2Fnursrep15010017&partnerID=40&md5=8cc1bc11b1c9ce2ec150a250032eb471

DOCUMENT TYPE: Review
OPEN ACCESS: ALL OPEN ACCESS; GOLD OPEN ACCESS; GREEN ACCEPTED OPEN ACCESS; GREEN OPEN ACCESS

Number of included studies: 26

26.
Herrera Jerez, M.J., Castro-Peraza, M.E., Delgado Morales, N.M., Arias-Rodríguez, A.
AUTHOR FULL NAMES: Herrera Jerez, M. J. (59500169900); Castro-Peraza, María Elisa (28367562900); Delgado Morales, N. M. (59500711900); Arias-Rodríguez, Maria De Los Ángeles (7101851668)
59500169900; 28367562900; 59500711900; 7101851668
Use of Hormone Blockers in Transgender Teenagers: A Scoping Review
(2024) Nursing Reports, 14 (4), pp. 4109 - 4118
DOI: 10.3390/nursrep14040299
https://www.scopus.com/inward/record.uri?eid=2-s2.0-85214112278&doi=10.3390%2Fnursrep14040299&partnerID=40&md5=4827c44d40a75e22584739d7d7a7388f

DOCUMENT TYPE: Review
OPEN ACCESS: ALL OPEN ACCESS; GOLD OPEN ACCESS; GREEN ACCEPTED OPEN ACCESS; GREEN OPEN ACCESS

Number of included studies: 6

27.

Antolí-Jover, A.M., Gázquez-López, M., Brieba-del Río, P., Pérez-Morente, M.Á., Martín-Salvador, A., Álvarez-Serrano, M.A.
AUTHOR FULL NAMES: Antolí-Jover, Ana María (57220046136); Gázquez-López, María (56381584200); Brieba-del Río, Pascual (57204560606); Pérez-Morente, María Ángeles (57041254400); Martín-Salvador, Adelina (56491031400); Álvarez-Serrano, María Adelaida (57193881468)
57220046136; 56381584200; 57204560606; 57041254400; 56491031400; 57193881468
Impact of Work–Family Balance on Nurses' Perceived Quality of Life During the COVID-19 Pandemic: A Scoping Review
(2024) Nursing Reports, 14 (4), pp. 4022 - 4038
DOI: 10.3390/nursrep14040294
https://www.scopus.com/inward/record.uri?eid=2-s2.0-85213485980&doi=10.3390%2Fnursrep14040294&partnerID=40&md5=3ea689e60ae4b486bd1cc4e88c27859b

DOCUMENT TYPE: Review
OPEN ACCESS: ALL OPEN ACCESS; GOLD OPEN ACCESS; GREEN ACCEPTED OPEN ACCESS; GREEN OPEN ACCESS

Number of included studies: 5

28.
Henderson, E., McConnell, H., Mitchell, G.
AUTHOR FULL NAMES: Henderson, Elizabeth (59362200000); McConnell, Hannah (59171914100); Mitchell, Gary (55641165800)
59362200000; 59171914100; 55641165800
Therapeutic Doll Interventions for People Living with Dementia in Care Homes: A Scoping Review
(2024) Nursing Reports, 14 (4), pp. 2706 - 2718
DOI: 10.3390/nursrep14040200
https://www.scopus.com/inward/record.uri?eid=2-s2.0-85213466672&doi=10.3390%2Fnursrep14040200&partnerID=40&md5=b81484021d3338d2868d37fa2319bf07

DOCUMENT TYPE: Review
OPEN ACCESS: ALL OPEN ACCESS; GOLD OPEN ACCESS; GREEN ACCEPTED OPEN ACCESS; GREEN OPEN ACCESS

Number of included studies: 12

29.
Timko Olson, E.R., Olson, A., Driscoll, M., Bliss, D.Z.
AUTHOR FULL NAMES: Timko Olson, Erica R. (6507833775); Olson, Anthony A. (58099975900); Driscoll, Megan (58099542600); Bliss, Donna Zimmaro (7102454924)
6507833775; 58099975900; 58099542600; 7102454924

Psychosocial Factors Affecting Wellbeing and Sources of Support of Young Adult Cancer Survivors: A Scoping Review
(2024) Nursing Reports, 14 (4), pp. 4006 - 4021
DOI: 10.3390/nursrep14040293
https://www.scopus.com/inward/record.uri?eid=2-s2.0-85213453101&doi=10.3390%2Fnursrep14040293&partnerID=40&md5=98652f13b12aa19d1b3c08e10dc85c20

DOCUMENT TYPE: Review
OPEN ACCESS: ALL OPEN ACCESS; GOLD OPEN ACCESS; GREEN FINAL OPEN ACCESS; GREEN OPEN ACCESS

Number of included studies: 13

30.
Rosa, D., Ingrande, L., Marcomini, I., Poliani, A., Villa, G., Sodano, M., Manara, D.F.
AUTHOR FULL NAMES: Rosa, Debora (57204830687); Ingrande, Laura (59492820500); Marcomini, Ilaria (57223038007); Poliani, Andrea (58312728300); Villa, Giulia (54584457800); Sodano, Martina (59492676200); Manara, Duilio Fiorenzo (6603110582)
57204830687; 59492820500; 57223038007; 58312728300; 54584457800; 59492676200; 6603110582
Perceived Pain in People Living with Amyotrophic Lateral Sclerosis—A Scoping Review
(2024) Nursing Reports, 14 (4), pp. 3023 - 3039
DOI: 10.3390/nursrep14040220
https://www.scopus.com/inward/record.uri?eid=2-s2.0-85213449745&doi=10.3390%2Fnursrep14040220&partnerID=40&md5=c041904ce447a7802cef21757e3c1ba1

DOCUMENT TYPE: Review
OPEN ACCESS: ALL OPEN ACCESS; GOLD OPEN ACCESS; GREEN ACCEPTED OPEN ACCESS; GREEN OPEN ACCESS

Number of included studies: 13

31.
Taneva, D.I., Gyurova-Kancheva, V.T., Kirkova-Bogdanova, A.G., Paskaleva, D.A., Zlatanova, Y.T.
AUTHOR FULL NAMES: Taneva, Daniela Ivova (57217157245); Gyurova-Kancheva, Vasilka Todorova (58484447600); Kirkova-Bogdanova, Angelina (57210209203); Paskaleva, Diana Angelova (57203288271); Zlatanova, Yovka Tinkova (59492822000)
57217157245; 58484447600; 57210209203; 57203288271; 59492822000
Electronic Nursing Records: Importance for Nursing and Benefits of Implementation in Health Information Systems—A Scoping Review
(2024) Nursing Reports, 14 (4), pp. 3585 - 3605
DOI: 10.3390/nursrep14040262

https://www.scopus.com/inward/record.uri?eid=2-s2.0-85213424158&doi=10.3390%2Fnursrep14040262&partnerID=40&md5=db0c1d821565bac1005bae0bd885319f

DOCUMENT TYPE: Review
OPEN ACCESS: ALL OPEN ACCESS; GOLD OPEN ACCESS; GREEN ACCEPTED OPEN ACCESS; GREEN OPEN ACCESS

Number of included studies: 36

32.
Ventura-Silva, J., Martins, M.M., Trindade, L.D.L., Faria, A.D.C.A., Pereira, S., Zuge, S.S., Pimenta Lopes Ribeiro, O.M.P.L.
AUTHOR FULL NAMES: Ventura-Silva, João Miguel Almeida (57454283400); Martins, Maria Manuela Ferreira Pereira Da Silva (58793229100); Trindade, Letícia De Lima (16069683500); Faria, Ana Da Conceição Alves (57201850720); Pereira, Soraia Cristina (57221865368); Zuge, Samuel Spiegelberg (57192413917); Pimenta Lopes Ribeiro, Olga Maria (55964342500)
57454283400; 58793229100; 16069683500; 57201850720; 57221865368; 57192413917; 55964342500
Artificial Intelligence in the Organization of Nursing Care: A Scoping Review
(2024) Nursing Reports, 14 (4), pp. 2733 - 2745
DOI: 10.3390/nursrep14040202
https://www.scopus.com/inward/record.uri?eid=2-s2.0-85207733756&doi=10.3390%2Fnursrep14040202&partnerID=40&md5=f912d70e15706bc8522fae534ee47c9a

DOCUMENT TYPE: Review
OPEN ACCESS: ALL OPEN ACCESS; GOLD OPEN ACCESS; GREEN ACCEPTED OPEN ACCESS; GREEN OPEN ACCESS

Number of included studies: 10

33.
Rodríguez-Álvaro, M., Brito-Brito, P.R., García Hernández, A.M., Galdona-Luis, I., Rodríguez-Suárez, C.A.
AUTHOR FULL NAMES: Rodríguez-Álvaro, Martín (26639823700); Brito-Brito, Pedro Ruymán (55207317100); García Hernández, Alfonso Miguel (26639299600); Galdona-Luis, Irayma (58246122100); Rodríguez-Suárez, Claudio Alberto (57213145259)
26639823700; 55207317100; 26639299600; 58246122100; 57213145259
Nursing Interventions in Primary Care for the Management of Maladaptive Grief: A Scoping Review
(2024) Nursing Reports, 14 (3), pp. 2398 - 2414
DOI: 10.3390/nursrep14030178

https://www.scopus.com/inward/record.uri?eid=2-s2.0-85205233936&doi=10.3390%2Fnursrep14030178&partnerID=40&md5=926a1ed43d06d8d712e68f8868063b55

DOCUMENT TYPE: Review
OPEN ACCESS: ALL OPEN ACCESS; GOLD OPEN ACCESS; GREEN ACCEPTED OPEN ACCESS; GREEN OPEN ACCESS

Number of included studies: 10

34.
Mukomafhedzi, N., Tshitangano, T., Tshivhase, S.
AUTHOR FULL NAMES: Mukomafhedzi, Ndivhuwo (58635624700); Tshitangano, Takalani Grace (56574235100); Tshivhase, Shonisani Elizabeth (57212392505)
58635624700; 56574235100; 57212392505
Exploring Intervention Frameworks to Improve Utilization of Elimination of Mother-to-Child Transmission Services in Africa: A Scoping Review
(2024) Nursing Reports, 14 (3), pp. 2580 - 2595
DOI: 10.3390/nursrep14030190
https://www.scopus.com/inward/record.uri?eid=2-s2.0-85205128200&doi=10.3390%2Fnursrep14030190&partnerID=40&md5=1b74b7a6e2120c0ef2dc1ac576fd2d66

DOCUMENT TYPE: Review
OPEN ACCESS: ALL OPEN ACCESS; GOLD OPEN ACCESS; GREEN ACCEPTED OPEN ACCESS; GREEN OPEN ACCESS

Number of included studies: 14

35.
Teixeira, G., Lucas, P., Gaspar, F.
AUTHOR FULL NAMES: Teixeira, Gisela (57927353000); Lucas, Pedro Ricardo Martins Bernardes (57218794026); Gaspar, Maria Filomena Mendes (56099744200)
57927353000; 57218794026; 56099744200
Impact of Nurse Manager’s Attributes on Multi-Cultural Nursing Teams: A Scoping Review
(2024) Nursing Reports, 14 (3), pp. 1676 - 1692
DOI: 10.3390/nursrep14030125
https://www.scopus.com/inward/record.uri?eid=2-s2.0-85205079972&doi=10.3390%2Fnursrep14030125&partnerID=40&md5=c9f4b1eecf2651945a8fc35cfbc603e2

DOCUMENT TYPE: Review
OPEN ACCESS: ALL OPEN ACCESS; GOLD OPEN ACCESS; GREEN ACCEPTED OPEN ACCESS; GREEN OPEN ACCESS

Number of included studies: 39

36.
Encinas-Monge, C., Hidalgo-Fuentes, S., Cejalvo, E., Martí-Vilar, M.
AUTHOR FULL NAMES: Encinas-Monge, Celia (59345186500); Hidalgo-Fuentes, Sergio (57203993398); Cejalvo, Elena (57375057000); Martí-Vilar, Manuel (55175578400)
59345186500; 57203993398; 57375057000; 55175578400
Interventions to Relieve the Burden on Informal Caregivers of Older People with Dementia: A Scoping Review
(2024) Nursing Reports, 14 (3), pp. 2535 - 2549
DOI: 10.3390/nursrep14030187
https://www.scopus.com/inward/record.uri?eid=2-s2.0-85205079695&doi=10.3390%2Fnursrep14030187&partnerID=40&md5=88f8024acef06ad88b6dfa04817b8404

DOCUMENT TYPE: Review
OPEN ACCESS: ALL OPEN ACCESS; GOLD OPEN ACCESS; GREEN ACCEPTED OPEN ACCESS; GREEN OPEN ACCESS

Number of included studies: 26

37.
Coelho, F., Furtado, L., Mendonça, N., Soares, H., Duarte, H., Costeira, C., Santos, C., Pereira Sousa, J.
AUTHOR FULL NAMES: Coelho, Fábio (57980053300); Furtado, Luís (36547782700); Mendonça, Natália (58625939100); Soares, Hélia Maria (25722333800); Duarte, Hugo Miguel Santos (57277177100); Costeira, Cristina Raquel Batista (57200547379); Santos, Cátia (57942540000); Pereira Sousa, Joana (57210926095)
57980053300; 36547782700; 58625939100; 25722333800; 57277177100; 57200547379; 57942540000; 57210926095
Predisposing Factors to Medication Errors by Nurses and Prevention Strategies: A Scoping Review of Recent Literature
(2024) Nursing Reports, 14 (3), pp. 1553 - 1569
DOI: 10.3390/nursrep14030117
https://www.scopus.com/inward/record.uri?eid=2-s2.0-85205074129&doi=10.3390%2Fnursrep14030117&partnerID=40&md5=cc015bd6306eb8c2e7f46a503d416936

DOCUMENT TYPE: Review
OPEN ACCESS: ALL OPEN ACCESS; GOLD OPEN ACCESS; GREEN ACCEPTED OPEN ACCESS; GREEN OPEN ACCESS

Number of included studies: 16

38.
Kim, J.-E., Jung, S.-O.
AUTHOR FULL NAMES: Kim, Jung-eun (58411115000); Jung, Sunok (57214691064)
58411115000; 57214691064
Correlates and Health Issues among Older Korean Immigrants Living Alone in the United States: A Scoping Review
(2024) Nursing Reports, 14 (3), pp. 1859 - 1870
DOI: 10.3390/nursrep14030139
https://www.scopus.com/inward/record.uri?eid=2-s2.0-85205045980&doi=10.3390%2Fnursrep14030139&partnerID=40&md5=4b0f650173bb74b1dea6ea084f0e7bd4

DOCUMENT TYPE: Review
OPEN ACCESS: ALL OPEN ACCESS; GOLD OPEN ACCESS; GREEN ACCEPTED OPEN ACCESS; GREEN OPEN ACCESS

Number of included studies: 12

39.
Dias, A., Araújo, B., Jesus, É.
AUTHOR FULL NAMES: Dias, António (58940973000); Araújo, Beatriz (57193525277); Jesus, Élvio Henriques (23495330300)
58940973000; 57193525277; 23495330300
Mapping and Characterizing Instruments for Assessing Family Nurses’ Workload: Scoping Review
(2024) Nursing Reports, 14 (3), pp. 2020 - 2029
DOI: 10.3390/nursrep14030151
https://www.scopus.com/inward/record.uri?eid=2-s2.0-85205038087&doi=10.3390%2Fnursrep14030151&partnerID=40&md5=0827dca2ead563e49f1252986b23951c

DOCUMENT TYPE: Review
OPEN ACCESS: ALL OPEN ACCESS; GOLD OPEN ACCESS; GREEN ACCEPTED OPEN ACCESS; GREEN FINAL OPEN ACCESS; GREEN OPEN ACCESS

Number of included studies: 14

40.
Tzeng, H.-M., Franks, H., Passy, E.
AUTHOR FULL NAMES: Tzeng, Huey Ming (hm) (7004870067); Franks, Hannah E. (59196579000); Passy, Elise (59197001100)
7004870067; 59196579000; 59197001100
Facilitators and Barriers to Implementing the 4Ms Framework of Age-Friendly Health Systems: A Scoping Review
(2024) Nursing Reports, 14 (2), pp. 913 - 930

DOI: 10.3390/nursrep14020070
https://www.scopus.com/inward/record.uri?eid=2-s2.0-85197183160&doi=10.3390%2Fnursrep14020070&partnerID=40&md5=0e937e9e89a26a3402a6487e27e4221b

DOCUMENT TYPE: Review
OPEN ACCESS: ALL OPEN ACCESS; GOLD OPEN ACCESS; GREEN FINAL OPEN ACCESS; GREEN OPEN ACCESS

Number of included studies: 19

41.
Medina Cruz, E., Palenzuela-Luis, N., Rodríguez-Novo, N., González-Suárez, M., Casas Hernández, R., Novo-Muñoz, M.M.
AUTHOR FULL NAMES: Medina Cruz, Erik (59196796300); Palenzuela-Luis, Natacha (57218137757); Rodríguez-Novo, Natalia (57195837024); González-Suárez, Miriam (56245702400); Casas Hernández, Raquel (59196173800); Novo-Muñoz, Maria Mercedes (57206178251)
59196796300; 57218137757; 57195837024; 56245702400; 59196173800; 57206178251
Instruments for Evaluating the Nutritional Status of Cancer Patients Undergoing Antineoplastic Treatment: A Scoping Review
(2024) Nursing Reports, 14 (2), pp. 1312 - 1323
DOI: 10.3390/nursrep14020099
https://www.scopus.com/inward/record.uri?eid=2-s2.0-85197162385&doi=10.3390%2Fnursrep14020099&partnerID=40&md5=a07932ec6cc0e4729f9cf820936630c7

DOCUMENT TYPE: Review
OPEN ACCESS: ALL OPEN ACCESS; GOLD OPEN ACCESS; GREEN ACCEPTED OPEN ACCESS; GREEN OPEN ACCESS

Number of included studies: 10

42.
López-Luis, N.L., Rodríguez-Álvarez, C., Cuellar-Pompa, L., Arias-Rodríguez, A.
AUTHOR FULL NAMES: López-Luis, Noelia (58960674700); Rodríguez-Álvarez, Cristobalina (6603647012); Cuellar-Pompa, Leticia (56001766000); Arias-Rodríguez, Maria De Los Ángeles (7101851668)
58960674700; 6603647012; 56001766000; 7101851668
Evaluation of Delayed Effective Discharge for Non-Medical Reasons in Patients Admitted to Acute Care Hospitals in Spain: A Scoping Review
(2024) Nursing Reports, 14 (1), pp. 12 - 24
DOI: 10.3390/nursrep14010002

https://www.scopus.com/inward/record.uri?eid=2-s2.0-85188959279&doi=10.3390%2Fnursrep14010002&partnerID=40&md5=e02294a3e954bea111c23331c5b6987d

DOCUMENT TYPE: Review
OPEN ACCESS: ALL OPEN ACCESS; GOLD OPEN ACCESS; GREEN ACCEPTED OPEN ACCESS; GREEN OPEN ACCESS

Number of included studies: 13

43.
Azevedo, R., Manuel, T., Alves, P.
AUTHOR FULL NAMES: Azevedo, Rita (58960886800); Manuel, Tania (57553441400); Alves, Paulo Jorge Pereira (57222710330)
58960886800; 57553441400; 57222710330
Non-invasive Ventilation Interventions for Skin Injury Prevention: Scoping Review
(2024) Nursing Reports, 14 (1), pp. 56 - 65
DOI: 10.3390/nursrep14010005
https://www.scopus.com/inward/record.uri?eid=2-s2.0-85188944888&doi=10.3390%2Fnursrep14010005&partnerID=40&md5=c65fe4ea5ceb16764441e70fa6c6f12e

DOCUMENT TYPE: Review
OPEN ACCESS: ALL OPEN ACCESS; GOLD OPEN ACCESS; GREEN FINAL OPEN ACCESS; GREEN OPEN ACCESS

Number of included studies: 11

44.
Reedy, N., Luyke, T., McGregor, R., King, R., Dawson, R., Robinson, B., Terry, D.
AUTHOR FULL NAMES: Reedy, Natasha (55385720200); Luyke, Patricia (57870117000); McGregor, Rowena (57188652122); King, Rachel (55797666500); Dawson, Rhonda (55363468700); Robinson, Brendon (59713461700); Terry, Daniel R. (54404152900)
55385720200; 57870117000; 57188652122; 55797666500; 55363468700; 59713461700; 54404152900
Men in Mental Health: A Scoping Review of Challenges, Contributions, and Future Possibilities of Recruiting into Nursing
(2025) Nursing Reports, 15 (3), art. no. 97, Cited 1 times.
DOI: 10.3390/nursrep15030097
https://www.scopus.com/inward/record.uri?eid=2-s2.0-105001371166&doi=10.3390%2Fnursrep15030097&partnerID=40&md5=c58f5cf1f5005742e1e467c7f087c80a

DOCUMENT TYPE: Review

PUBLICATION STAGE: Final
OPEN ACCESS: ALL OPEN ACCESS; GOLD OPEN ACCESS; GREEN ACCEPTED OPEN ACCESS; GREEN OPEN ACCESS

Number of included studies: 24

45.
Gaspar, L., Reis, N., Sousa, P., Paiva E Silva, A.P.E., Cardoso, A., Brito, A., Bastos, F., Campos, J., Parente, P., Pereira, F., Machado, N.
AUTHOR FULL NAMES: Gaspar, Luís Jorge (58181061100); Reis, Neuza (58026860900); Sousa, Paula Cristina Moreira Mesquita De (57226149438); Paiva E Silva, Abel Avelino De (37038296600); Cardoso, Alexandrina Maria Ramos (7101743208); Brito, Alice (59235314000); Bastos, Fernanda Dos Santos (57190250251); Campos, Maria Joana (36695906100); Parente, Paulo Gonçalves (57222042992); Pereira, Filipe Miguel Soares (36466747800); Machado, Natália De Jesus Barbosa (57430256700)
58181061100; 58026860900; 57226149438; 37038296600; 7101743208; 59235314000; 57190250251; 36695906100; 57222042992; 36466747800; 57430256700
Nursing Process Related to the Nursing Focus “Airway Clearance”: A Scoping Review
(2024) Nursing Reports, 14 (3), pp. 1871 - 1896, Cited 0 times.
DOI: 10.3390/nursrep14030140
https://www.scopus.com/inward/record.uri?eid=2-s2.0-85205260589&doi=10.3390%2Fnursrep14030140&partnerID=40&md5=33d39d74427aab27f56f072f8a1784a2

DOCUMENT TYPE: Review
PUBLICATION STAGE: Final
OPEN ACCESS: ALL OPEN ACCESS; GOLD OPEN ACCESS; GREEN ACCEPTED OPEN ACCESS; GREEN OPEN ACCESS

Number of included studies: 123

Supplementary Table S1, key differences between scoping and systematic reviews

	Scoping Review	Systematic Review
**Purpose**	Exploratory mapping of a broad topic. Aims to identify key concepts, types of evidence, and knowledge gaps.	Addresses a specific question (often about intervention safety and effectiveness). Seeks to generative a definitive confidence (GRADE) statement to inform policy and practice
**Review formulation**	Broad review question often framed using the PCC (Population–Concept–Context) framework.	Focused review question typically structured using PICO (Population, Intervention, Comparator, Outcome) framework. Question framed to determine the safety and effectiveness of an intervention.
**Protocol development**	Recommended but not necessary. Historically have not required formal registration although this seems to be shifting. Protocol defines objectives, study eligibility criteria, and review methods.	A detailed protocol is considered standard practice, generally following PRISMA-P (21). Protocol includes review question, specific inclusion criteria, search strategy, and analysis methods. Typically, the protocol is registered (PROSPERO, Open Science Framework)
**Inclusion criteria**	Broad and potentially iterative. Developed to capture all relevant outputs around topic not restrictive by study design. Criteria may be further refined as research increase familiarity with the evidence base. Studies not excluded based on quality.	Narrow, pre-specified and locked. Inclusion criteria relate to interventions, outcomes, and study design. Criteria strictly applied during title and abstract and full text screening that is usually completed by at least two researchers independently.
**Data extraction**	Data are "charted" using a form to qualitatively extract relevant information from each included output. Charting focuses on capturing the descriptive characteristics (e.g. population, context, methodology, findings) related to the concept or question. Quality appraisal of studies is not required.	Data extraction to collect necessary data to address the review question. Typically, this includes sample size, intervention details, outcomes (events, mean differences, effect sizes). Data extraction completed by two researchers. Quality appraisal - completed by two researchers - determined using standardized measure (e.g. RoB2 (22)).
**Synthesis Methods**	Descriptive or narrative synthesis. Research collate and summarize findings qualitatively or using simple quantitative counts. Findings presented as a narrative summary of themes with the aim of providing an overview of what we know about a	Meta-analysis to address the review question. Where meta-analysis is not possible a structured narrative synthesis focused on the outcomes of interest. Quality appraisal may be used to inform subgroup analyses or sensitivity analyses excluding studies with high risk of bias. Alternatively,

	topic (e.g. number and type of studies).	quality appraisal used to make an informed statement on confidence of evidence to support the application of an intervention.
**Impact**	Provides justification of full systematic review or gaps in knowledge that warrant further investigation. No impact on policy or clinical practice.	Provides a sound rational for the inclusion of intervention as part of standard of care.
**Reporting guidelines**	PRISMA-ScR and JBI guidelines.	PRISMA (2020)
